# P2X7 receptor and NLRP3 inflammasome activation in head and neck cancer

**DOI:** 10.18632/oncotarget.16903

**Published:** 2017-04-06

**Authors:** Ju Young Bae, Sang-Woo Lee, Yong-Hwan Shin, Jong-Ho Lee, Jeong Won Jahng, Kyungpyo Park

**Affiliations:** ^1^ Department of Physiology, School of Dentistry, Seoul National University, Seoul 110–749, Korea; ^2^ Oral and Maxillofacial Surgery, School of Dentistry, Seoul National University, Seoul 110–749, Korea; ^3^ Dental Research Institute, Seoul 110–749, Korea

**Keywords:** purinergic receptor P2X7, NLRP3 inflammasome, head and neck squamous cell carcinoma, A253 cells, invasiveness

## Abstract

In this study, we investigated purinergic receptor P2X7 and *NACHT*, *LRR* and *PYD domains-containing protein 3* (NLRP3) inflammasome expressions, and their role in head and neck cancer. We found upregulation of purinergic receptor P2X7 and all NLRP3 inflammasome components in biopsied head and neck squamous cell carcinoma tissues. Similarly, the expression of purinergic receptor P2X7, apoptosis-associated speck-like protein containing CARD, and pro-form caspase 1 in A253 cells derived from epidermoid carcinoma were highly upregulated in comparison to normal Human Salivary Gland cell line. Active caspase-1 and its final product, active interleukin-1β, both increased in primed A253 cells stimulated with purinergic receptor P2X7 agonists, while this elevated NLRP3 inflammasome activity was suppressed by purinergic receptor P2X7 antagonists. However, we observed none of these effects in Human Salivary Gland cells. Inhibition of both NLRP3 inflammasome and purinergic receptor P2X7 led to the significant cell death of primed A253 cells, but had no effect on the viability of primed HSG cells or the primary cultured human fibroblast cells. Furthermore, inhibition of either purinergic receptor P2X7 or NLRP3 inflammasome decreased invasiveness of A253, and this effect became more evident when both purinergic receptor P2X7 and NLRP3 inflammasome were simultaneously blocked. Therefore, it is concluded that the purinergic receptor P2X7 and the activation of NLRP3 inflammasome play important roles in the survival and invasiveness of head and neck squamous cell carcinoma in humans.

## INTRODUCTION

The purinergic receptor P2X7 (P2X7R), a member of the P2XR subfamily, is known to be triggered by ATP molecules to open its gate, mediating Ca^2+^ influx and K^+^ ion efflux. Generally, P2X7R is known as a cytotoxic channel, since its sustained activation creates non-selective pores on cell membranes, leading to cell death [1]. However, recent studies have suggested the possibility that P2X7R facilitates the survival and invasion of tumor cells. For example, P2X7R-transfected cells showed increased growth activity and invasive activity both *in vivo* and *in vitro* [2]. Over expression of P2X7R has also been reported in various types of cancer, such as leukemia, neuroblastoma, melanoma, prostate, breast, and thyroid cancer [3–5]. Several mechanisms and pathways have been suggested to understand the functional roles of P2X7 in cancer.

The NLRP3 inflammasome pathway is considered to be one of the most important P2XTR-induced downstream pathways [6]. According to previous studies, the NLRP3 inflammasome is assembled when ATP activates P2X7R to efflux K+ ions [7]. NLRP3 inflammasome is a protein complex composed of NOD-like receptor protein 3 (NLRP3), apoptosis-associated speck-like protein containing a CARD (ASC), and caspase-1. Fully assembled NLRP3 inflammasome cleaves pro-form caspase-1(p45) into active caspase-1(p20), and the active caspase-1 sequentially cleaves pro interleukin-1β (IL-1β) into active IL-1β, which is then released to extracellular space [7–9].

Previous studies have reported that either immune or non-immune cells under certain pathologic conditions such as Sjögren's syndrome [10], Bechet's disease [11], Type 2 diabetes [12], rheumatoid arthritis [13], and various types of cancers [2, 4, 14–18] show elevated expression of P2XR and/or NLRP3 inflammasome. Extensive studies have been conducted on P2XR and/or NLRP3 inflammasome in colon cancer [17], melanoma [19], prostate cancer [18], and lung cancer [15], but only a few studies has been done in the field of Head and Neck Squamous Cell Carcinoma (HNSCC). Mann et al. reported that proinflammatory cytokines including IL-1, -4, and -6 were detected in HNSCC [20] and Chen et al. found that the HNSCC patients showing high serum level of IL-1a, IL-6, IL-8, GM-CSF, and VEGF cytokines generally had a poor prognosis [14]. However the exact functional role or production mechanism of these proinflammatory cytokines in HNSCC has not been identified. In this study, we propose that the interaction between overexpressed functional P2X7R and NLRP3 components is crucial to proinflammatory cytokine production and to the survival and invasiveness of HNSCC.

## RESULTS

### Over-expression of P2X7R in oral cancer tissue

**Figure 1:**
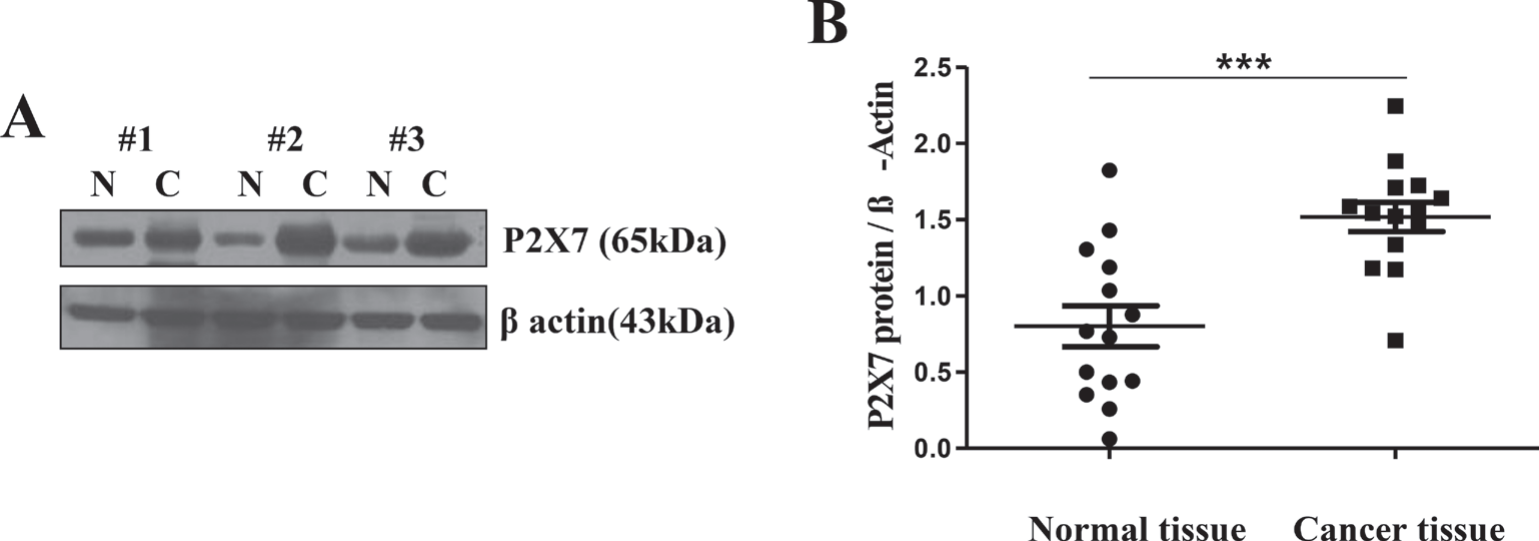
Over-expression of P2X7R in oral cancer tissue (**A**) Excised HNSCC and normal tissues were collected from three different patients to assess the expression level of P2X7R by Western blot (Lanes 1, 3, 5: normal tissues; lanes 2, 4, 6: oral cancer tissues). (**B**) The expression levels of P2X7R were significantly higher in the HNSCC patient group than in the control group (***P < 0.001, *n* = 14).

First, we examined head and neck cancer tissues collected from six different patients to measure levels of P2X7R expression. All of the samples were histologically diagnosed as squamous cell carcinoma (SCC). (Figure 1A) shows the representative Western blot analyses from three patients. P2X7R expression was upregulated compared to that of the normal tissues. (Figure 1B) shows a summarized result. The expression levels of P2X7R were significantly higher in the patient group than in the control group. (*P* = 0.0002, *n* = 14)

### Over-expression of inflammasome components in oral cancer tissue

**Figure 2:**
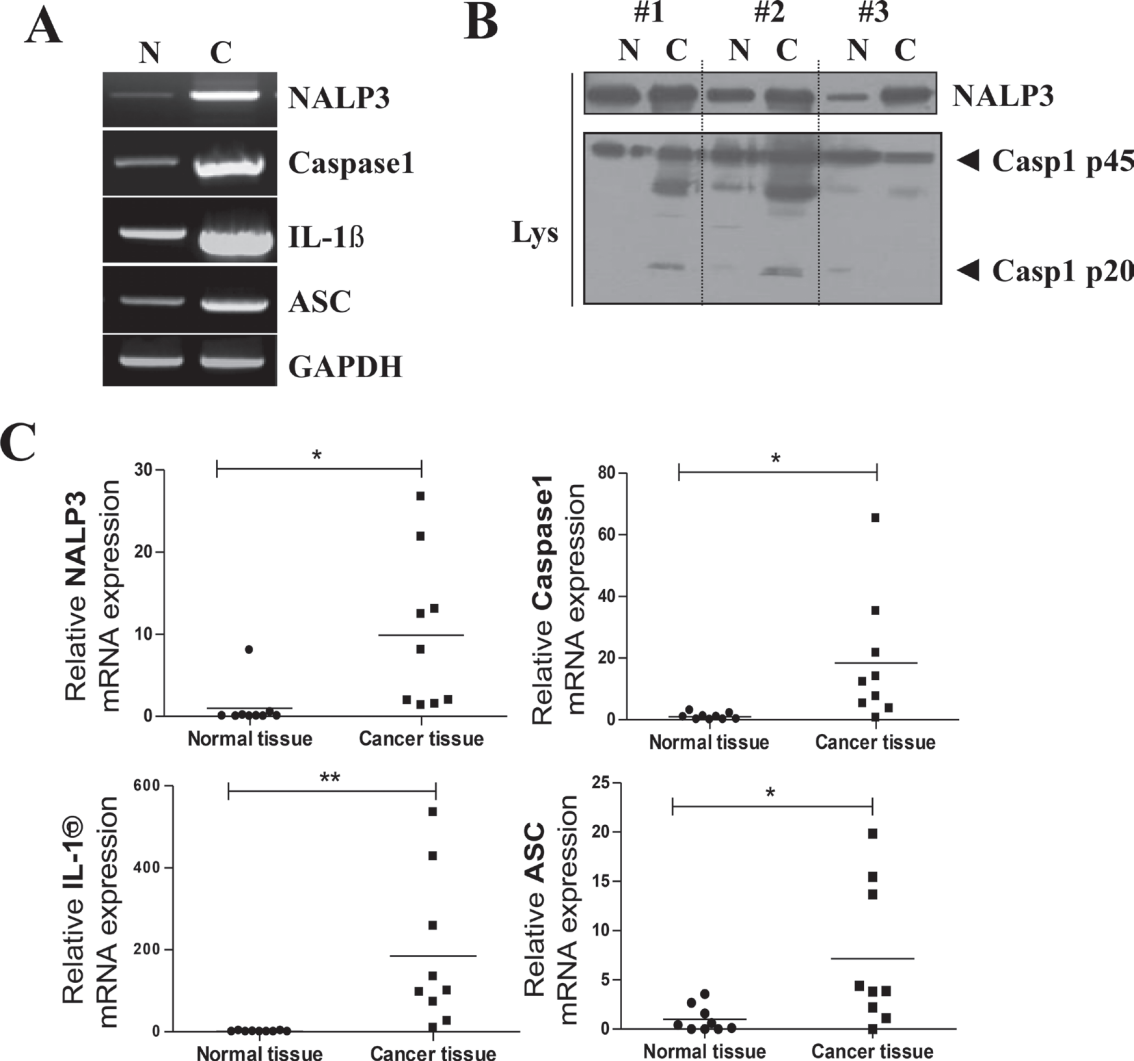
Over-expression of inflammasome components in oral cancer tissue (**A**) mRNA expression of the inflammasome components NLRP3, caspase-1, and ASC and the inflammatory cytokines IL-1β. GAPDH is shown as a loading control. (**B**) A representative Western blot of three normal human tissue (N) and three HNSCC samples shows the protein expressions of NLRP3 protein, pro-caspase-1 (45 kDa), and activated caspase-1 p20 (20 kDa). (**C**) qPCR data collected from nine HNSCC patients' tumor and normal tissues. Raw data from normal tissues of each group was averaged and set as 1, thus fold change of each individual sample based on this value was plotted (*n* = 9, *P < 0.05, **P < 0.01).

We also found significant upregulation of the mRNA levels of NLRP3, ASC, caspase-1, and IL-1β in the HNSCC mass (NLRP3; *P* = 0.0153, Caspase-1; *P* = 0.0216, IL-1β; *P* = 0.0087, ASC; *P* = 0.0225, *n* = 9) (Figure 2A, 2C). NLRP3 inflammasome activity was also determined by measuring the protein level of active caspase-1 p20 (20 kDa). As expected, active caspase-1 was robustly detected in HNSCC tissues, while it was rarely detected in normal tissues (Figure 2B). Although the mRNA levels of NLRP3 inflammasome components were generally elevated in HNSCC mass, the degree of its elevation was very different in each patient. We compared the various tumor properties and prognosis of each patient and found correlation(s) with degrees of NLRP3 inflammasome overexpression. Five of the nine HNSCC patients subjected to qPCR analysis consented to our review of their medical history chart and surgery report. We averaged the mRNA expression level fold changes of ASC, IL-1β, Caspase-1, and NLRP3 for each patient, and aligned the results from low to high average fold change value. (Table 1) shows an association between patients' prognosis and tumor mass property and the expression level of P2X7R and NALP3 inflammasome. We found that the patient with lowest average value showed no recurrence, metastasis, and no specific post-operative problems. In contrast, patient No.5 at maximum average value showed an extremely bad prognosis with recurrence, multiple distant metastasis, inflammatory changes in Sternocleidomastoid muscle (SCM m.) post-surgery, and largest tumor size with deepest depth of invasion among the five patients. Patients number 2,3, and 4 showed either recurrence or metastasis but showed little differences in prognosis, tumor size, and depth of invasion.

**Table 1:** Various tumor properties and prognosis of each patient and correlation(s) with degrees of NLRP3 inflammasome overexpression

Patient number	Patient age/sex	mRNA expression fold change (HNSCC/Normal tissue)				Average fold change	Recurrence	Metastasis	Tumor size (cm3)	Depth of invasion (cm)	Objective findings after surgery
		ASC	IL-1b	Caspase1	NLRP3						
1	75/F	1.69872	0.267326	0.721025	9.765946	3.113254	No	No	17.64	2.1	N/S
2	67/F	7.434229	14.20622	1.605869	1.392034	6.159587	No	Yes (1/28 LN)	26.64	1.2	N/S
3	60/M	7.561513	33.04599	5.781747	1.72429	12.02839	Yes	No	7.92	1.5	Right neck level II recur 1Y 2M after surgery No distant metastasis
4	58/M	23.02545	77.49942	0.990481	12.26093	28.44407	No	Yes (2/33 LN)	7.392	1.2	N/S
5	58/M	368.354	16035.55	3516.315	21734.22	10413.61	Yes	Yes (Multiple, distant metastasis)	35.84	3.2	Recur 1Y7M after surgeryLt neck, mediastinum Left SCM m. swelling Inflammatory change Metastatic lymph node, multiple: left level Ib, Iia, III, IV

Averaged mRNA expression level fold changes of ASC, IL-1β, Caspase-1, and NLRP3 for each patient is aligned from low to high average fold change value. History of recurrence and metastasis is analyzed, and tumor size (cm3) and depth of invasion (cm) recorded in pathophysiology lab reports and surgery reports are described. Post-surgery medical reports are collected, and any abnormal or significant post-surgery events are described as “objective findings after surgery”. Five patients were analyzed.

### Functional upregulation of P2X7R and NLRP3 inflammasome components in A253 cells

**Figure 3:**
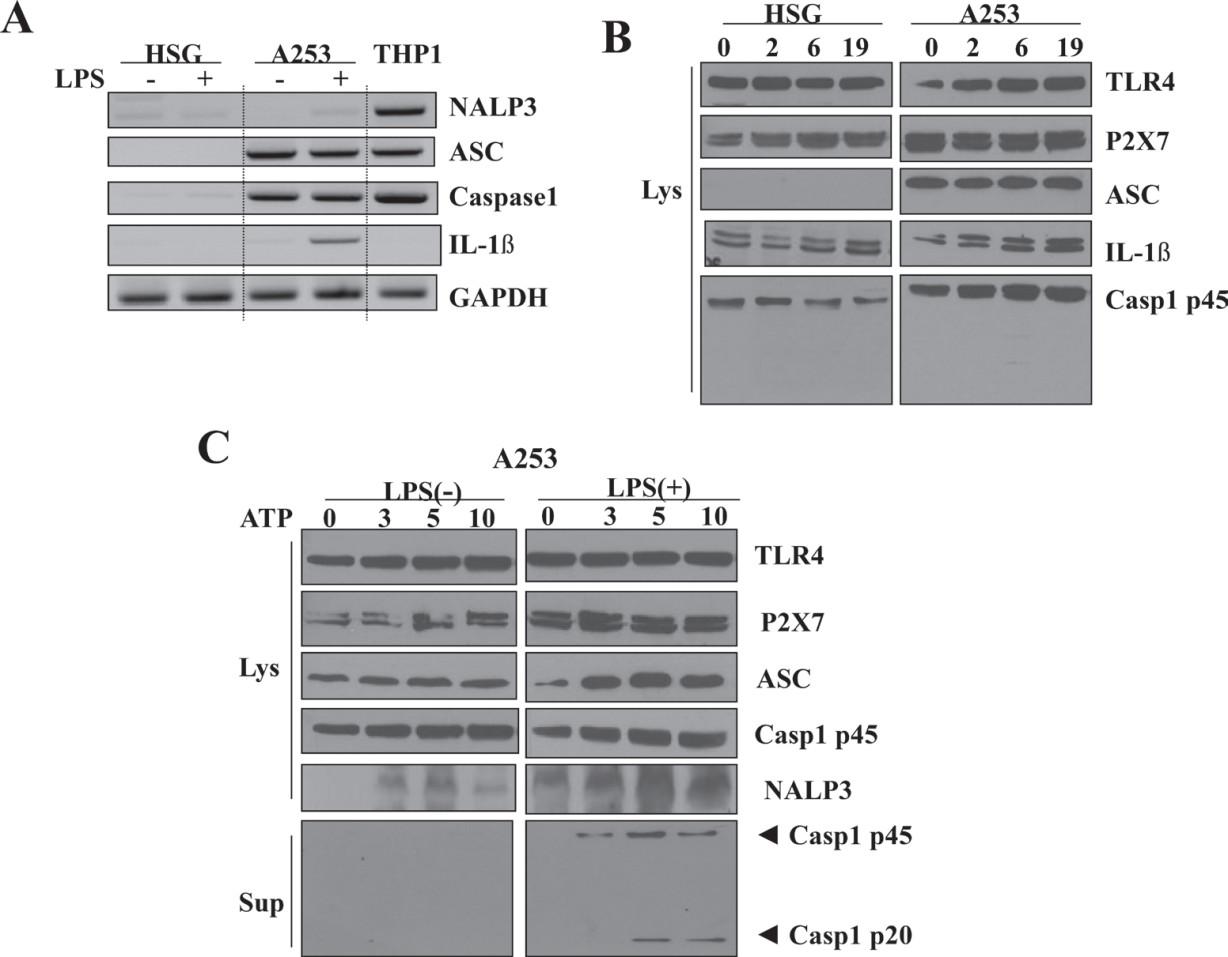
Upregulation of P2X7R and NLRP3 inflammasome components in A253 cells (**A**) The mRNA expression of NLRP3 inflammasome components in A253 and HSG cells. THP-1 cells, a macrophage cell line, were used as a positive control. Cells were treated with LPS for priming. RT-PCR analysis shows detectable levels of mRNA coding for members of the NLRP3 inflammasome complex. Results shown are representative of 3–4 individual experiments. (**B**) Expression of NLRP3 inflammasome in HSG and A253 cells at 0, 2, 6, and 19 h after stimulation with 1 μg/ml LPS. (**C**) NLRP3 inflammasome signaling is dispensable for ATP dose-dependent caspase-1 activation induced by LPS in A253 cells.

The over-expression of P2X7R and NLRP3 inflammasome components in cancer tissues was further observed through using two types of salivary gland epithelial cell lines: Human Salivary Gland (HSG) cells derived from the duct of human submandibular glands and A253 cells derived from the epidermoid carcinoma harvested from human submandibular glands. P2X7R and the components related to NLRP3 inflammasome were upregulated in the A253 cells compared to the HSG cells (Figure 3).

Before we examined the functional relationship between P2X7R and NLRP3 inflammasome, the cells were pretreated with LPS for priming. After the stimulation of HSG, A253, and THP-1 (human monocytic cell line) cells with 1 μg/ml LPS, the mRNA levels of inflammasome components including ASC, caspase-1, and NLRP3 were examined. THP-1 cells were used as a positive control group (Figure 3A). In both LPS-primed and unprimed HSG cells, the mRNA of NLRP3 inflammasome components was not detected. In contrast, in the A253 cells, expressions of ASC and procaspase-1 were increased. Intracellular pro-IL-1β was significantly increased in the primed A253 cells, but no mature IL-1β was detected, since there was no detectable caspase-1 p20. P2X7R, TLR4, and procaspase-1 were also expressed in HSG cells, but their expression levels were much less than those in the A253 cells, and no significant expressional change was observed after LPS stimulation. In contrast to the A253 cells, ASC expression was not detected in HSG cells at all. Although pro-IL-1β expression was slightly increased by LPS treatment in HSG cells, caspase-1 p20 and mature IL-1β were not detected (Figure 3B).

However, when A253 cells were stimulated with ATP, all components of the NLRP3 inflammasome (including ASC, procaspase-1 (45kDa), and NLRP3) gradually increased in proportion to ATP concentration. Caspase-1 p20 became detectable after the priming step at ATP concentrations above 5 mM. Next, we examined whether LPS stimulation is required to activate the NLRP3 inflammasome via P2X7R activation. When A253 cells were stimulated with ATP alone, caspase-1 p20 was not detected, and no change in the expression levels of the P2X7R and NLRP3 components was observed by increasing ATP concentration. However, when A253 cells were stimulated with ATP after the LPS priming step, all components of the NLRP3 inflammasome (ASC, procaspase-1 (45 kDa), and NLRP3) gradually increased in proportion to the ATP concentration, and caspase-1 p20 also became detectable at ATP concentrations above 5 mM (Figure 3C).

### Role of P2X7R in the activation of NLRP3 inflammasome

**Figure 4:**
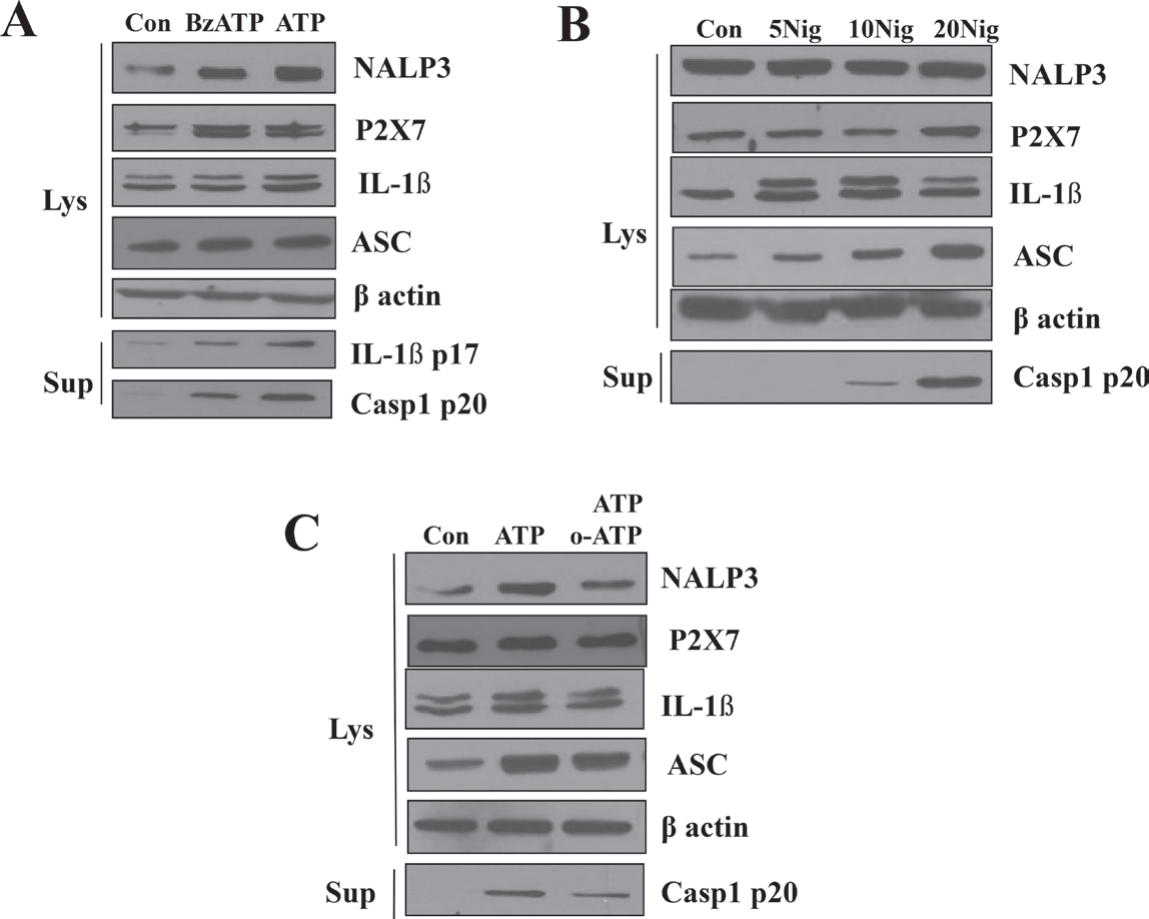
Role of P2X7R in the activation of NLRP3 inflammasome (**A**) LPS-primed A253 cells were stimulated with 0.3 mM 2,3-O-(4-benzoyl-benzoyl)ATP (BzATP) for 3 h except for ATP that was added 30 min before the end of the experiment. Treatment with 5 mM ATP or 0.3 mM BzATP induced the release of caspase-1. (**B**) A253 cells were stimulated for 6 h with the indicated amounts (per ml) of nigericin. The range of nigericin concentrations was from 5 μM to 20 μM. Results shown are representative of 3–4 individual experiments (**C**) ATP or oxATP induced the release of caspase-1 in LPS-primed A253 cells.

We further examined whether the NLRP3 inflammasome is activated by a specific P2X7R agonist in the presence or the absence of the antagonist. When primed A253 cells were treated with 0.3 mM 3-O-benzoylbenzoicacid-derivatized ATP analogue (BzATP; a specific agonist of P2X7R), the expressions of P2X7R itself, NLRP3, and pro-IL-1β were significantly increased to similar levels as when treated with 5 mM ATP concentrations. Active-form IL-1β (17kDa) and caspase-1 p20 were also detected when primed A532 cells were treated with 0.3 mM BzATP or 5mM ATP (Figure 4A). We obtained a similar result with another P2X7R agonist, nigericin. As the concentration of nigericin increased from 5 μM to 20 μM, ASC expression levels also gradually increased. Furthermore, active caspase-1 (p20) became detectable at the nigericin concentration of 10 μM and increased as the nigericin concentration rose to 20 μM (Figure 4B). Next, we tested oATP, a specific blocker of P2X7R. Compared to the groups treated only with ATP, the groups treated with both oATP and ATP showed decreased expression of NLRP3, but still showed higher degrees of expression than that of control groups (Figure 4C). The decrease in the protein level of active caspase-1 p20, a key enzyme for IL-1β maturation, affected by oATP clearly indicates that NLRP3 inflammasome activation is mediated by P2X7R (Figure 4C).

### Activity of P2X7R and NLRP3 inflammasome is closely related to the survival and invasiveness of A253 cells

**Figure 5:**
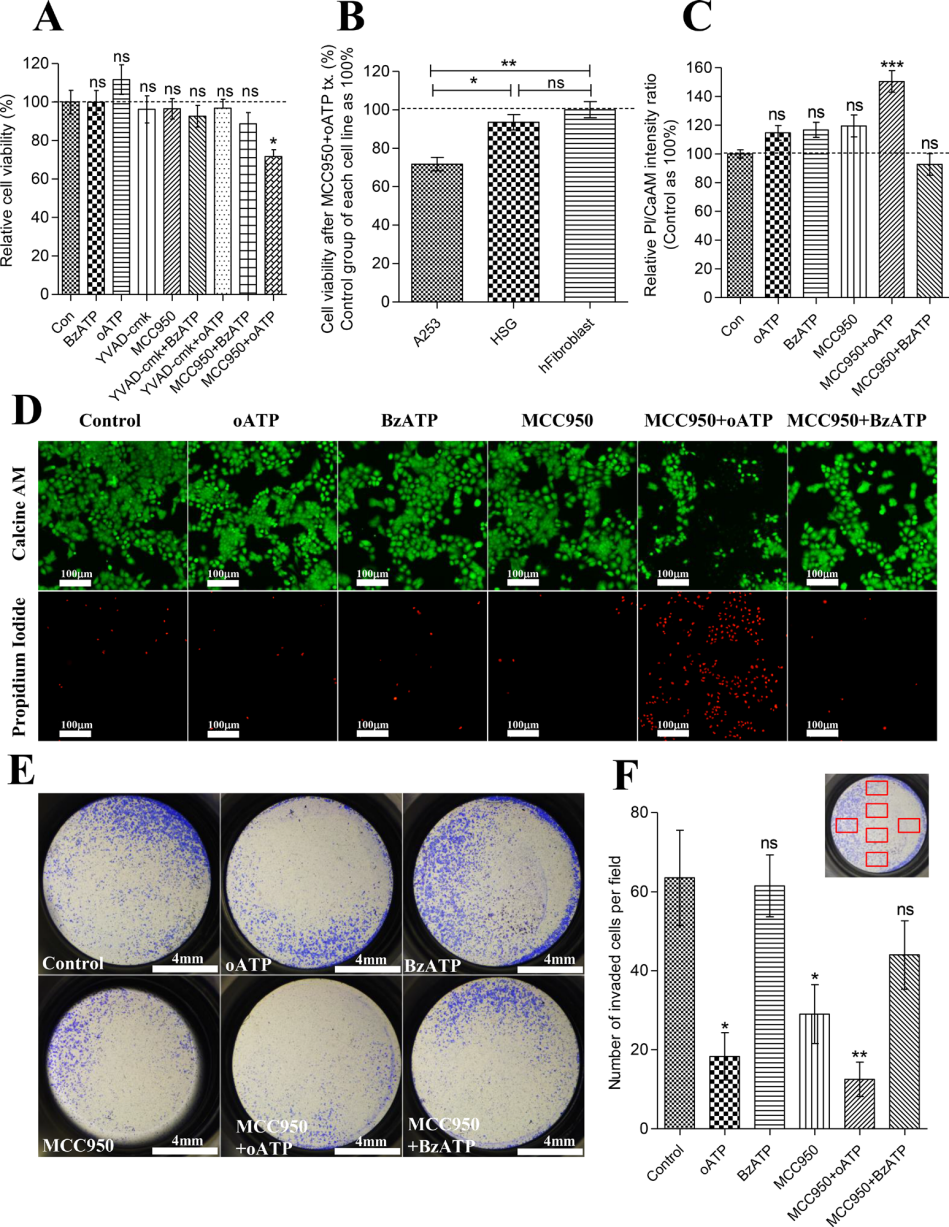
Activity of P2X7R and NLRP3 inflammasome is closely related to the survival and invasiveness of A253 cells (**A**) CCK-8 cell viability assay of LPS-primed A253 cells treated with oATP (1 μM), BzATP (0.3 mM), caspase-1 inhibitor (Ac-YVAD-cmk; 30 μg/ml), MCC950 (10 μM), or in combination of one another for 24hrs. Absorbance values at 450nm of control group were averaged and set as 100%. Data are expressed as the mean ± SEM of *n* = 6 (*P = < 0.05). (**B**) CCK-8 cell viability assay of LPS-primed A253, HSG, and primary cultured human fibroblast cells treated with combination of oATP (1 mM)+MCC950(10 μM). Absorbance values at 450 nm of each cell line's control group were averaged and set as 100%. Data are expressed as the mean ± SEM of *n* = 6 (*P = < 0.05, **P < 0.01). (**C**) Relative fluorescence intensity ratio of Propodium Iodide/Calcein AM. LPS-primed A253 cells were treated with oATP (1 mM), BzATP (0.3 mM), MCC950 (10 μM), or in combination of one another for 24 hrs. FL intensity ratios of PI/CaAM of control group were averaged and set as 100%. Data are expressed as the mean ± SEM of *n* = 6 (*P = < 0.05, **P < 0.01). (**D**) Epifluorescence image of PI/CaAM. LPS-primed A253 cells were treated with oATP (1 mM), BzATP (0.3 mM), MCC950 (10 μM), or in combination of one another for 24 hrs. FL images of PI channel were equally enhanced by increasing 40% brightness and 40% contrast. FL images of CaAM are unmodified images. (**E**) Matrigel invasion assay result. LPS-primed A253 cells were treated with oATP (1 mM), BzATP (0.3 mM), MCC950 (10 μM), or in combination for 24 hrs and trypsinized. 50000 viable cells were reseeded in each insert of the invasion chamber. 24 hrs later, invaded cells were fixed and stained with 1% crystal violet, and images of entire well were captured. 3 independent experiments were carried out. (**F**) Number of invaded cells per 20× magnified field was manually counted. Six different area of insert were selected and captured by Inverted microscope with 20X magnification. Selected areas are described in the graph as red squares. Data are expressed as the mean ± SEM of *n* = 6 (*P = < 0.05, **P < 0.01).

We next examined the biological roles of P2X7R and NLRP3 inflammasome in A253 cells. Antagonist (oATP; 20 μM) and agonist (BzATP; 0.3 mM) of P2X7R and specific inhibitor of NLRP3 inflammasome (MCC950; 10 μM) were used independently or in combination. MCC950 was developed by Rebecca C et al. and is known to specifically inhibit both canonical and noncanonical NALRP3 activation by blocking NLRP3-induced ASC oligomerization [21]. MCC950 does not block AIM2, NLRC4, TLR pathways nor the K+ efflux, Ca2+ efflux, or NLRP3-ASC interaction, which makes it ideal to independently examine NLRP3 and P2X7 in LPS-primed cells [21]. A253 cells were LPS-primed for 19 hrs and then treated with oATP (1 mM), BzATP (0.3 mM), Ac-YVAD-cmk (caspase-1 inhibitor; 30 μg/ml), MCC950 (NLRP3 inflammasome inhibitor; 10 μM), or in combination of one another for 24 hrs. CCK8 cell viability assay results showed significant decrease in cellular respiratory rate only when P2X7R and NLRP3 inflammasome were both inhibited by oATP and MCC950 in A253 cells (*P* = 0.0117, *n* = 6) (Figure 5A). However, there was no significant reduction of cell viability when 30 μg/ml of Ac-YVAD-cmk, a caspase-1 inhibitor, was treated with oATP/BzATP on A253 cells (Figure 5A). Significant increase in viability over control group was not observed in all groups (Figure 5A). This decrease in the cell viability by MCC950+oATP treatment was not observed in normal cells including HSG and primary cultured human fibroblast cells from a section of biopsied sample histologically diagnosed as normal (Figure 5B). To find out whether this decreased viability rate in A253 cells was due to cell death or declined proliferative capacity we performed Propidium iodide(PI)/Calcein AM dead or live assay. Significantly increased infiltration of PI dye and decreased metabolized Calcein AM signals in MCC950+oATP treated A253 cells (*P* = 0.0006, *n* = 6) indicated that the inhibition of both P2X7R and NLRP3 inflammasome induced actual cell death (Figure 5C, 5D). Next we examined the role of P2X7R and NLRP3 inflammasome in invasiveness of A253 cells. To minimize the cell death induced by MCC950+oATP during invasion process, 24 hr-drug-treated A253 cells were trypsinized and an equal number of viable cells was seeded to upper chamber matrigel filled with pure DMEM. 24hrs later invaded cells were stained with crystal violet and manually counted in 20X magnified view. Invasiveness of A253 cells were significantly reduced by blocking either P2X7R or NLRP3 inflammasome (oATP; *P* = 0.0162, MCC950; *P* = 0.0323, *n* = 6) (Figure 5E, 5F). However the reduction of invasiveness became more evident when both P2X7R and NLRP3 inflammasome were inhibited (MCC950+oATP; *P* = 0.0033, *n* = 6) (Figure 5E, 5F).

## DISCUSSION

We describe here that P2X7R and NLRP3 inflammasome, over-expressed in HNSCC, are potentially related to the survival and invasiveness of HNSCC cells *in vitro*, and further, to the prognosis of HNSCC patients.

We found that both P2X7R and NLRP3 inflammasome component expression were upregulated in cancer tissues compared to normal tissues. Active caspase-1 p20 was detected in every HNSCC cancer tissue sample, suggesting that IL-1β also has to be cleaved to become activated. Since active caspase-1 was detected in HNSCC samples, it is evident that HNSCC can produce active IL-1β by itself. However, *in vitro* experiments revealed that a very small amount of active IL-1β was released extracellularly from A253 (epithelial cells) compared to immune cells such as THP-1 cell. Therefore, we suspected that the release of active IL-1β from HNSCC cells was due to pyroptosis, which could be induced by Gasdermin D activation via active caspase-11 or caspase -1, rather than general exocytosis mechanism [22]. Chen et al. also reported that IL-1β was not detected in patients' serum [14]. Although limited amounts of released active IL-1β cannot affect distant areas around tumor mass, it may have affected tumor mass itself as an autocrine signal or microenvironment to enhance its malignancy. In tumor immunology, IL-1 is associated with cancer progression and development [6]. IL-1 and -6 are usually produced by tumor-associated macrophages such as the M2 macrophage. However, our findings suggest that the stimulation of over-expressed P2X7R on HNSCC or epithelial cells produces active IL-1β via the NLRP3 inflammasome pathway independent of the surrounding tumor-associated macrophages. Several studies have reported that the tumor microenvironment contains high concentrations of IL-1, and that it promotes the angiogenesis and proliferation of tumor cells via indirect stimulation of neighboring normal cells to produce angiogenic and metastatic factors [16]. In fact, when we matched degree of mRNA expression data of NLRP3, ASC, IL-1β, and caspase-1 with each patient's medical records, increased tendencies to recur and metastasize were observed with increased expression levels. Similar phenomena have been reported in studies on the relationship between IL-1 and prognosis of solid tumors including breast, colon, lung, and melanoma [16]. Due to limited number of patients it is difficult to statistically prove this hypothesis in our study, but further clinical studies may reveal more detailed correlations between NLRP3 inflammasome pathway and HNSCC malignancy.

We also confirmed that ATP-mediated activation of P2X7R is capable of inducing inflammasome activation in the HNSCC A253 cell line. In unstimulated HSG cells, NLRP3 components were barely detected at either mRNA or protein level. However, in the A253 cells, these components were constitutively upregulated without any stimulation. P2X7R expression and its activity were also significantly elevated in A253 cells compared to HSG cells. This result is consistent with our previous study reporting that CpG islands of P2X7R gene are naturally hypermethylated in HSG cells [23]. The specific stimulation of P2X7R by BzATP activated the NLRP3 inflammasome pathway in A253 cells, but interestingly, neither P2X7R nor NLRP3 inflammasome activation was detected in HSG cells even after ATP or BzATP stimulation following the priming step. It seems that the P2X7R expressed in HSG cells are in a somewhat “inactive” state unresponsive to the agonists. Our previous study also showed that A P2X7R-induced Ca^2+^ increase was not detected in HSG cells even after treatment with high concentration of ATP or BzATP [23]. Therefore, it is highly likely that the NLRP3 inflammasome pathway is generally silenced in normal epithelial cells; however, when they become cancerous, the NLRP3 inflammasome becomes activated by the increased expression and activity of P2X7R.

Therefore, we suspected that targeting P2X7R and NLRP3 inflammasome may mitigate HNSCC malignancy without harming normal tissues surrounding them. Consistent with this premise, significant cell death was observed only when P2X7R and NLRP3 inflammasome were both inhibited by oATP and MCC950, a specific inhibitor of NLRP3 inflammasome. However treatment of a caspase-1 inhibitor with oATP or BzATP did not induce significant cell death. This indicates that caspase-1 or its final product, IL-1β, is not crucial for HNSCC cell survival in isolated conditions without a surrounding tumor microenvironment. In addition the MCC950+oATP combination did not induce cell death in normal cells which neither overexpress P2X7R nor NLRP3 inflammasome components. This result shows a possibility that the MCC950+oATP combination can be therapeutically used to specifically kill HNSCC without harming surrounding normal cells. Invasiveness of A253 was also largely suppressed by P2X7R and NLRP3 inflammasome blockers. We found that an inhibition of either P2X7R or NLRP3 inflammasome was enough to reduce invasiveness of A253, although reduction of invasiveness became more evident when both were blocked.

Based on our results, it seems survival of P2X7R/NLRP3 inflammasome-over-expressing cells is largely dependent on the caspase-1 independent NLRP3 inflammasome pathway. Several studies have reported that NLRP3 and ASC have caspase-1-independent function. ASC by itself, independent of caspase-1, can induce autoimmune diseases including arthritis and encephalitis [24, 25]. Wang et al. reported that NLRP3 and ASC caspase-1-independently regulate Transforming growth factor- β1 (TGF- β1) in renal tubular epithelial cells [26]. Considering that the TGF- β1 promotes cancer invasion and metastasis via enhancement of EMT related genes, it is possible that this mechanism is behind reduced invasion by P2X7R and NLRP3 inhibition. However, only a few mechanism studies have been conducted to explain caspase-1-independent NLRP3/ASC activities. Further investigations will be required to unveil the mechanism of reduced invasion and viability caused by simultaneous inhibition of P2X7R and NLRP3.

In this study, we suggested that P2X7R and NLRP3 inflammasome are over-expressed in HNSCC and that the degree of expressional level may represent prognosis of patients. To our knowledge, this is the first report that HNSCC can produce active IL-1β via P2X7/NLRP3 inflammasome pathways, and also the first trial to reduce HNSCC survival and invasiveness by blocking P2X7R/NLRP3 inflammasome. These findings on the relationship between P2XR and NLRP3 inflammasome in HNSCC may contribute to the development of novel therapeutic approaches or diagnostic markers predicting the prognosis and degree of HNSCC malignancy.

## MATERIALS AND METHODS

### Reagents

Lipopolysaccharide (LPS; *Escherichia coli* 0111:B4), ATP, and oxidized ATP (oxATP) were obtained from Sigma (St. Louis, MO, USA), and nigericin was purchased from InvivoGen (San Diego, CA, USA). Ac-YVAD-cmk and MCC950(CP-456773 sodium salt) were purchased from Sigma (St. Louis, MO, USA). Cell Counting Kit-8 (CCK-8) was purchased from Dojindo (Japan). Calcein AM iwas purchased from Invitrogen (Carlsbad, CA, USA) and 1 mg/ml Propidium iodide solution was purchased from Sigma (St. Louis, MO, USA). 1% Crystal violet solution was purchased from Sigma (St. Louis, MO, USA). NLRP3 antibodies and secondary anti-sheep antibody conjugated to horseradish peroxidase (HRP) were purchased from R&D Systems (Minneapolis, MN, USA). Toll-like receptor 4 (TLR4), P2X7, ASC, IL-1β, and caspase-1 donkey anti-rabbit IgG-HRP were purchased from Santa Cruz Biotechnology (Santa Cruz, CA, USA).

### Cell culture

HSG cells isolated from human submandibular ducts and A253 cells derived from human squamous carcinoma in submandibular glands were cultured in Dulbecco's Modified Eagle's Medium (Welgene, Daegu, South Korea) supplemented with 10% fetal bovine serum (Welgene) and 1% penicillin/streptomycin (Gibco, Carlsbad, CA, USA) at 37°C in a humidified 5% CO_2_ incubator. Media change was done three times a week and sub-cultured when cells were approximately 80% confluent. 0.25% trypsin/1.0 mM ethylenediaminetetraacetic acid (Welgene) was used to detach cells from the culture dish. Primary cultured normal human fibroblast cells were obtained from biopsy sample of a patient. Normal tissue was submerged in 70% EtOH for 2 min for the sterilization and washed twice with PBS. Normal tissue was minced in HBSS and transferred to cell culture dish containing DMEM supplemented with 10% FBS and 1% penicillin/streptomycin, and incubated for 7 days. At the day 7 media and unattached cells were completely removed. Attached cells were split into four 100mm dishes and continuously subcultured or cryopreserved. Media was changed twice a week.

### RT-PCR

Total RNA from normal submandibular gland tissues and oral squamous cell carcinoma tissues were extracted using Trizol (Invitrogen, Carlsbad, CA, USA) according to the manufacturer's protocol. The extracted total RNA concentration was quantified using a NanoDrop^®^ ND-2000 spectrophotometer (NanoDrop Technologies, Wilmington, DE, USA). Reverse transcriptase (Invitrogen) was used with 1 μg of total RNA. Polymerase chain reaction (PCR) with specific primers was performed using 1 μl of cDNA.

The primer sets were as follows: NLRP3: forward 5′-ATG GCA AGC ACC CGC TGC-3′ and reverse 5′-GCT GTC TTC CTG GCA TAT CAC AG-3′; ASC: forward 5′-GCC AAG CCA GGC CTG CAC-3′ and reverse 5′-GCT CCG CTC CAG GTC CTC-3′; caspase-1: forward 5′-GAA ACA AAA GTC GGC AGA GA-3′ and reverse 5′-TGG GAA GAG GTA GAA ACA TC-3′; IL-1β: forward 5′-ATG GCA GAA GTA CCT AAG CTC GC-3′ and reverse 5′-ACA CAA ATT GCA TGG TGA AGT CAG TT-3′; and GAPDH: forward 5′-TTC ACC ACC ATG GAG AAG GC-3′ and reverse 5′-TCA TGA CCA CAG TCC ATG CC-3′. PCR conditions were as follows: 35 cycles of denaturation at 95°C for 55 s, annealing at 55°C for 30 s, and extension at 72°C for 55 s; and a final step at 72°C for 10 min. Products from RT-PCR reactions were sequenced to confirm their expression. The PCR products were confirmed by ethidium bromide staining after 1.5% agarose gel electrophoresis.

### Quantitative real-time PCR

Total RNA was isolated from Oral squamous cell carcinoma tissues and normal submandibular gland tissues using the Trizol reagent (Invitrogen), and then used in Superscript III reverse transcriptase reaction (Invitrogen) for cDNA synthesize with and oligo-(dT) primers according to the manufacturer's protocol. For quantitative Real-time PCRs were performed with SYBR green premix buffer (Applied Bio-Systems, Foster City, CA, USA) and an ABI Prism 7500 sequence detector (Applied Biosystems), and relative expression levels were determined after normalization to the threshold cycle (CT) values for GAPDH.

The gene-specific primers were as follows: for NLRP3: forward 5′- TCC TCA GCA GCA ACC AGA AG -3′ and reverse 5′- GCT TCA GTC CCA CAC ACA GA-3′; ASC: forward 5′- TCC TCA GTC GGC AGC CAA G -3′ and reverse 5′-TCA GGA CCT TCC CGT ACA GA -3′; Caspase-1: forward 5′- CAA GGT CCT GAA GGA GAA GAG A -3′ and reverse 5′- TGT TCA GCA CCC TTG TCT GT-3′; IL-1β: forward 5′- AGT ACC TGA GCT CGC CAG T-3′ and reverse 5′- CTG GAA GGA GCA CTT CAT CTG T-3′; and GAPDH: forward 5′- GTC AAG GCT GAG AAC GGG AA -3′ and reverse 5′- TGG ACT CCA CGA CGT ACT CA - 3′.

### Human tissue

Oral squamous cell carcinoma tissues and normal submandibular gland tissues were obtained from the patients. Biopsied tissues were stored in 4°C normal HEPES buffer right after the surgical excision for the further processing. Gland tissues obtained from the same patients were confirmed as non-cancerous by pathology department of SNU dental hospital and thus used as normal or control groups. All ethical guidelines and consent forms were approved by Institutional Review Board of Seoul National University Dental Hospital (CRI11023G).

### Western blot assay

After the three-times of phosphate-buffered saline washes cells were lysed by RIPA buffer supplemented with 1% protease inhibitor cocktail (GenDEPOT, Katy, TX, USA). Protein concentration was measured using the bicinchoninic acid protein assay kit (Pierce Biotechnology, Rockford, IL), and bovine serum albumin was used to generate protein concentration standard curve. Proteins were separated on 10% sodium dodecyl sulfate polyacrylamide gel electrophoresis (Bio-Rad, Hercules, CA, USA), and this was followed by electrotransfer onto nitrocellulose membranes (Whatman, Dassel, Germany). The membranes were blocked with 10% non-fat milk (Seoul-milk, Seoul, Korea) in Tris-buffered saline with Tween for 1 h, and the blots were then incubated overnight at 4°C with a primary anti-NLRP3 antibody (R&D Systems), anti-P2X7 antibody (Santa Cruz Biotechnology), anti-IL-1β antibody (Santa Cruz Biotechnology), anti-ASC antibody (Santa Cruz Biotechnology), anti-caspase-1 antibody (Santa Cruz Biotechnology), anti-TLR4 antibody (Santa Cruz Biotechnology), and anti-actin antibody (Santa Cruz Biotechnology) and were then incubated with HRP-conjugated secondary antibody (Santa Cruz Biotechnology). The bands were developed by an enhanced electrochemiluminescence detection system (Thermo Scientific, Waltham, MA, USA). The bindings of the specific antibodies were visualized using Agfa film (Agfa, Mortsel, Belgium).

### CCK-8 cell viability assay

Cells were seeded on 96-well plate with 5000cell/well density and incubated in 37°C in a humidified 5% CO_2_ incubator for 12 hrs. Then, media is changed to DMEM containing 1 μg/ml LPS, and cells were incubated for another 19 hrs for priming. After the priming step each group (*n* = 6) of cells were treated with oATP (1 mM), BzATP (0.3 mM), Ac-YVAD-cmk (30 μg/ml), MCC950 (10 μM), or in combination. 24hrs after the drug treatment media was changed to non-phenol red DMEM containing 5% CCK-8 stock solution. 1 hr later absorbance was measured at 450 nm in Synergy 2 microplate reader (Biotek).

### Propidium iodide/calcein AM live dead assay

A253 Cells were seeded on two black walled 96-well plate (SPL, Korea) with 5000cell/well density and incubated in 37°C in a humidified 5% CO_2_ incubator for 12 hrs. Then, media is changed to DMEM containing 1 μg/ml LPS, and cells were incubated for another 19 hrs for priming. After the priming step each group (*n* = 4 for imaging /*n* = 6 for FL measurement) of cells was treated with oATP (1 mM), BzATP (0.3 mM), Ac-YVAD-cmk (30 μg/ml), MCC950 (10 μM), or in combination. 24 hrs after the drug treatment media was changed to non-phenol red DMEM containing 3 μM Propidium iodide and 3 μM Calcein AM. After the 30 min of incubation images were obtained by Digital Inverted Fluorescence Microscope (Nikon) with 20× magnification. Another plate was subjected to Synergy 2 microplate reader (Biotek) to measure fluorescence intensity of PI (Ex: 540/25 Em: 590/20) and Calcein AM (Ex: 485/20 Em:528/20). PI fluorescence values were divided by Calcein AM fluorescence values to get the relative ratio of cell death FL signal out of viable cell signal.

### Matrigel invasion assay

A253 cells were seeded on six 100 mm cell culture dish with 1 × 10^6^ cells/dish density and incubated in 37°C in a humidified 5% CO_2_ incubator for 12 hrs. Then, media is changed to DMEM containing 1 μg/ml LPS, and cells were incubated for another 19 hrs for priming. After the priming step each group of cells was treated with oATP (1 mM), BzATP (0.3 mM), Ac-YVAD-cmk (30 μg/ml), MCC950 (10 μM), or in combination. 24 hrs after the drug treatment cells were washed and detached with 0.5% Trypsin and dispersed in DMEM and then transferred to upper chamber of pre-humidified 24-well Corning BioCoat Matrigel Invasion Chamber (#354480 8.0 μm PET membrane). Lower chamber was filled with 750 ul of 10% FBS containing DMEM to attract cells. After the 24 hrs of incubation each insert was washed twice with PBS and un-migrated cells were wiped away by using cotton sticks. Migrated cells were fixed with 4% Paraformaldehyde (PFA) for 10 min and stained with 1% Crystal violet solution for 30 min. Inserts were washed twice with tap water to remove remaining Crystal violet solution and completely dried up for 2 hrs in drying oven. Images of invaded cells were captured by Leica S6D stereo microscope with 2.5× magnification. Number of invaded cells was manually counted based on the images obtained at six different locations per group using Digital Inverted Fluorescence Microscope (Nikon) with 20× magnification.
